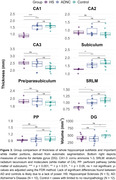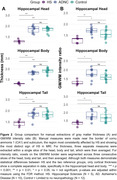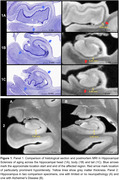# Postmortem MRI signature of Hippocampal Sclerosis of Aging

**DOI:** 10.1002/alz.093795

**Published:** 2025-01-09

**Authors:** Gustaf Rådman, Amanda E Denning, Sadhana Ravikumar, Nicola Spotorno, Ranjit Ittyerah, Lisa M Levorse, Madigan Bedard, Eunice Chung, John L. Robinson, Theresa Schuck, Eddie B Lee, Sydney A Lim, Winifred Trotman, Alejandra Bahena, Rosanna K Olsen, David J Irwin, Maria del Mar Arroyo Jimenez, Alicia Vela, Esther Buendia, Maria Mercedes Iniguez de Onzono Martin, Maria del Pilar Marcos Rabal, Monica Munoz, Ricardo Insausti, Paul A. Yushkevich, Laura E.M. Wisse

**Affiliations:** ^1^ Lund University, Lund Sweden; ^2^ University of Pennsylvania, Philadelphia, PA USA; ^3^ Rotman Research Institute, Toronto, ON Canada; ^4^ University of Castilla‐La Mancha, Albacete Spain

## Abstract

**Background:**

Hippocampal Sclerosis of aging (HS) refers to age‐related selective neuronal loss and gliosis in hippocampal cornu ammonis 1 (CA1) and subiculum that is out of proportion to tau pathology in Alzheimer’s Disease (AD). HS is related to cognitive decline and memory impairments separately from other neurodegenerative pathologies. To date, in vivo imaging biomarkers of HS of aging are non‐existent, and their development would greatly improve diagnosis and prognosis in memory clinics. We therefore aimed to characterize the magnetic resonance imaging (MRI) signature of HS among autopsy confirmed cases of HS.

**Method:**

Data consisted of same‐subject histological Nissl‐stained slices and postmortem MRI (0.2mm isotropic) acquired from 27 subjects at the University of Pennsylvania and University Castilla‐La Mancha. Based on neuropathological assessment, cases were grouped into HS (N = 5), intermediate to high AD neuropathologic change without HS (N = 10) and limited or no neuropathological burden (N = 12). We manually measured grey matter thickness and GM/WM intensity ratio in select locations across the hippocampal head, body, and tail. We also obtained more global structural measures from hippocampal subfields using an automated postmortem atlas‐based segmentation algorithm.

**Result:**

Qualitatively, HS is clearly noticeable as cortical thinning and hypointense signal (approximating white matter) on postmortem MRI across the CA1 and subiculum, particularly anteriorly closer to the border between CA1/subiculum (Figure 1). Manual thickness measures showed complete separation between the HS group and the two reference groups across both the hippocampal head and body, and a less pronounced difference in the tail. Global structural measures from automated segmentations corroborated these group differences showing complete separation in CA1 thickness and significant differences in subiculum and the white matter layer of CA1 (Stratum Radiatum Lacunosum Moleculare, SRLM).

**Conclusion:**

In MRI, HS is marked by hypointense voxels and cortical thinning across CA1 and subiculum. Both local manual thickness measurements and thickness measures of whole subfields derived from automatic segmentations provided promising results. As a next step, we plan to perform similar measurements on antemortem MRI of the same subjects, with the hopes of making progress toward an in vivo MRI biomarker of HS.